# Bridging the gaps in Universal Health Coverage using Digital Health Citizenship

**DOI:** 10.1371/journal.pdig.0000913

**Published:** 2025-07-10

**Authors:** Rupal Jain, Rajarshi Dasgupta

**Affiliations:** 1 Digital Transformations for Health Lab, Geneva, Switzerland; 2 Indian Institute of Technology, Delhi, India; McGill University, CANADA

## Introduction

Universal Health Coverage (UHC) has long been a global aspiration to achieve by 2030 under the Sustainable Development Goals (SDGs) target 3.8 (1). UHC aims to ensure that all individuals have access to the full range of quality health services, including prevention, treatment, rehabilitation, and even palliative care, without any kind of financial hardship. World Health Organization (WHO) data shares that about 4.5 billion people over the globe did not have complete access to essential health services in the year 2021 [1].

In recent years, Digital Health (DH) services have been increasingly recognized as a potential enabler of UHC. By leveraging technology, these services promise to expand access, reduce costs, and improve the quality of care [2]. However, the path to achieving this aim is filled with challenges [3]. Looking from a macro perspective, there are certain prerequisites for successful digital health adoption, which will be understood using Toyoma’s Theory of Technology as an Amplifier in this article.

## Technology as an amplifier

Toyama, in his influential work on technology and social change, proposed that technology acts merely as an amplifier of underlying human and institutional intent and capacity [4]. It was first developed under “information and communication technology for development” (ICT4D) and has been widely adopted due to its broad applicability in the development sector. It states that technology should be considered multiplicative in nature and not additive. In simpler words, technology does not add or disruptively transform society by itself; instead, it amplifies whatever is already present in society. Technology cannot replace human intent or ability where they are absent. It proposed three mechanisms of amplification: differential access, capacity, and intent.

Toyama’s theory serves not just as an analytical lens but as a strategic framework to guide policy design in digital health. Rather than viewing digital health failures as purely technical or infrastructural, this lens redirects attention to systemic social factors. In low- and middle-income countries (LMICs), including India, this means focusing efforts not only on technological rollouts but also on strengthening the ecosystem through education, trust-building, and participatory governance.

## Differential access: The risk of widening inequities

While digital health tools hold immense promise, their benefits are often shared disproportionately with those already advantaged, primarily individuals in higher-income countries (HICs) or higher socio-economic classes within LMICs. Rural areas, in particular, often lack the basic infrastructure needed to support digital health initiatives. Communities with limited access to smartphones, internet connectivity, or even electricity are left further behind, deepening the digital divide. For instance, during COVID-19, various Digital Platforms were launched within countries to contact tracing, navigate the hotspots, and identify red alert zones (e.g., Aarogya Setu in India, COVIDsafe in Australia, TousAntiCovid in France, and many more such applications) [5–7]. However, the usage of these applications was limited to only those who had access to digital technology.

## Differential capacity: Bridging through education

While universal allocation of technology can combat differential access. Disparities in education, income, and social skills can still result in unequal abilities to effectively use technology [8]. However, the solution may lie within the theory itself. According to theory, the three mechanisms of differential access, capacity, and intent are traditionally viewed as coexisting factors, nonetheless, it would be more effective to consider these as progressive stages, with one leading to the next.

## Intent/motivation: Call for stricter laws

Literature suggests that while most of the LMICs are still grappling with access and capacity, HICs are facing issues with intent/motivation [9,10]. Intent represents the willingness and motivation of individuals and systems to adopt and sustain these technologies. In simpler terms, “what people want to do with the technology they have access to” [4].

Based on the literature, trust stands out as a significant and persistent challenge in the adoption of DH services especially in HICs and among people with higher educational levels [11]. One such study mentioned, despite having access and capability to use the TousAntiCovid application for contact tracing in France, 63.3% of people never downloaded the application because of fear of misuse of personal data along with excessive battery consumption [7]. Issues such as concerns over data privacy, the credibility of digital health platforms, and ethical transparency have hindered the widespread acceptance of DH systems [12]. Unlike the above two mechanisms, where the policies need to be user-intensive, this issue is to be countered on the provider side with the introduction and implementation of stricter privacy and data security laws.

## Discussion

Digital Health holds significant potential to achieve UHC, but its success depends on more than mere technological innovation. Addressing the intertwined challenges of access, capacity, and intent requires a holistic approach like Digital Health Citizenship (DHC), which works at the intersection of Digital Literacy, Health Literacy, and Civic Literacy [13]. It includes informed decision-making, active participation, and ethical use of DH technologies and platforms.

DHC solves the challenges of access using targeted interventions like public participation-focused digital infrastructure development. It also brings attention to digital health education through capacity-building programs aiming to eliminate differential capacity, highlighted by Toyoma. Similarly, to address the concerns for differential motivation, which are based on safety and privacy issues, DHC advocates for strong digital governance and transparent policies, keeping the user at the center of the policies. It promotes enhancing user control over personal health data. Overall, DHC provides the roadmap for equitable access, improved literacy, and trust-building mechanisms essential for sustainable digital health integration.

To provide a structured roadmap for advancing digital health connectivity, it is essential to begin by prioritizing public investments in digital infrastructure, particularly in underserved rural and peri-urban areas. This can be effectively achieved through public–private partnerships, focusing on ensuring reliable internet access, stable electricity supply, and affordable digital devices. Equally important is the need to design user-friendly technologies, ensuring no one is left behind, by minimizing digital literacy barriers.

We also need to move away from treating health and education in silos. Instead, they should reinforce each other by designing digital health literacy campaigns as part of primary healthcare outreach. A core aim of DHC is to bridge the last-mile gap and help realize UHC. Yet, from what have been observed in many LMIC contexts, frontline health workers are not positioned at the forefront of digital health strategies. They must be seen as key enablers in the adoption and scaling of digital health technologies. This means training community health workers to act as digital health facilitators, helping to close capacity gaps and build trust at the community level.

The next step involves strengthening the ecosystem for adoption, which includes implementing strong, context-specific data protection laws. These should be modeled after global frameworks like the General Data Protection Regulation, emphasizing user consent, data privacy, and algorithmic transparency. Finally, it’s essential to recognize that no single policy will work across all contexts. Strategies must reflect the developmental stage, institutional readiness, and socio-cultural realities of each country.
